# A modified neural circuit framework for semantic memory retrieval with implications for circuit modulation to treat verbal retrieval deficits

**DOI:** 10.1002/brb3.3490

**Published:** 2024-04-29

**Authors:** Hsueh‐Sheng Chiang, Raksha A. Mudar, Christine S. Dugas, Michael A. Motes, Michael A. Kraut, John Hart

**Affiliations:** ^1^ Department of Neurology University of Texas Southwestern Medical Center Dallas Texas USA; ^2^ School of Behavioral and Brain Sciences The University of Texas at Dallas Richardson Texas USA; ^3^ Department of Speech and Hearing Science University of Illinois Urbana‐Champaign Champaign Illinois USA; ^4^ Department of Radiology and Radiological Science Johns Hopkins University Baltimore Maryland USA

**Keywords:** caudate, frontal aslant tract, fronto‐polar region, fronto‐striatal tract, left inferior frontal gyrus, pre‐Supplementary motor area, thalamus, verbal production, verbal retrieval, word finding

## Abstract

Word finding difficulty is a frequent complaint in older age and disease states, but treatment options are lacking for such verbal retrieval deficits. Better understanding of the neurophysiological and neuroanatomical basis of verbal retrieval function may inform effective interventions. In this article, we review the current evidence of a neural retrieval circuit central to verbal production, including words and semantic memory, that involves the pre‐supplementary motor area (pre‐SMA), striatum (particularly caudate nucleus), and thalamus. We aim to offer a modified neural circuit framework expanded upon a memory retrieval model proposed in 2013 by Hart et al., as evidence from electrophysiological, functional brain imaging, and noninvasive electrical brain stimulation studies have provided additional pieces of information that converge on a shared neural circuit for retrieval of memory and words. We propose that both the left inferior frontal gyrus and fronto‐polar regions should be included in the expanded circuit. All these regions have their respective functional roles during verbal retrieval, such as selection and inhibition during search, initiation and termination of search, maintenance of co‐activation across cortical regions, as well as final activation of the retrieved information. We will also highlight the structural connectivity from and to the pre‐SMA (e.g., frontal aslant tract and fronto‐striatal tract) that facilitates communication between the regions within this circuit. Finally, we will discuss how this circuit and its correlated activity may be affected by disease states and how this circuit may serve as a novel target engagement for neuromodulatory treatment of verbal retrieval deficits.

## Graphical abstract

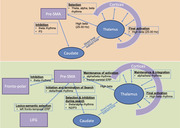

## 1 INTRODUCTION

Word finding difficulty is a common complaint in older age and is often reported in a variety of neurologic diseases (Rohrer et al., 2008). Clinical evaluation using various tasks (e.g., confrontation naming, category fluency, phonemic fluency, and picture description) is used to confirm and to characterize verbal retrieval deficits. Mechanisms underlying these verbal retrieval deficits may vary across diseases, hence rendering a symptom‐targeted therapy extremely challenging. In this article, based on neurophysiological and neuroanatomical evidence, we extend a previously postulated neurocognitive model centered on a neural circuit for semantic memory retrieval. This neural circuit may inform the development of new treatments for verbal retrieval deficits across neurological diseases.

**FIGURE 1:**
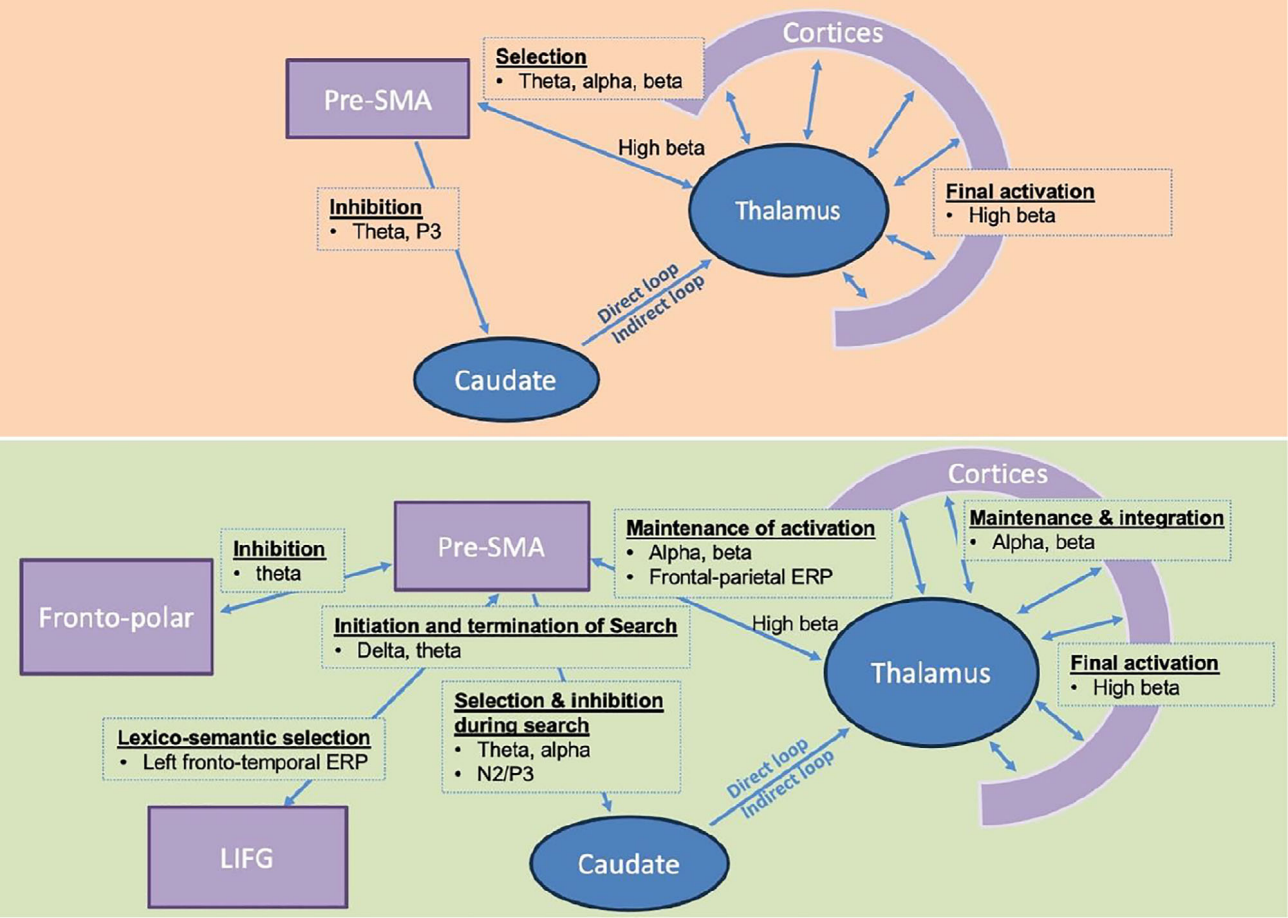
Hart et al. (2013) neural circuit model is depicted on the top; the current updated neurocognitive network framework is depicted at the bottom. See text for detailed description. Purple shade represents cortical regions; blue shad represents subcortical regions. LIFG, left inferior frontal gyrus; Pre‐SMA, pre‐supplementary motor area.

The retrieval function likely requires coordination between several different cognitive processes (see Text Box 1). Hart et al. (2013) proposed a neural circuit model for semantic memory retrieval based on evidence from studies conducted in healthy and clinical populations using functional magnetic resonance imaging (fMRI) and task‐based electroencephalography (EEG) during the Semantic Object Retrieval Test (SORT) and the semantic inhibition (SI) tasks (see Text Box 2). These two tests are designed to examine retrieval functions involved in semantic retrieval. In this model, the pre‐supplementary motor area (pre‐SMA) generates sustained theta‐band EEG activity that has been hypothesized to reflect controlled search and eventual selection processes (Figure 1; see Text Box 3 for electrophysiological studies for cognitive function). Activation and maintenance of the cortical representations of a concept such as that of an object is hypothesized to be accentuated and prolonged via a neural pathway extending from the caudate to thalamus, resulting in enhanced thalamocortical activity (Crosson, 2013; Crosson et al., 2009). An indirect caudate loop suppresses the activity in cortical representations of incorrect objects, leading to their non‐retrieval. These processes, including those involved in selection (theta, alpha, and beta rhythms) and inhibition (theta and activity around 300–500 ms post stimulus measured by P3 event‐related potential), may recur until the decision is made (Figure 1; see also Crosson et al., 2009). The processes reflecting the integration of features related to an object memory occurs in the left frontotemporal region around 750 ms post stimulus onset (Brier et al., 2008). As the cortical representations of multiple features of the correct object emerge and converge, there is interaction between the pre‐SMA, thalamus, and parieto‐occipital cortical regions. This interaction is indexed by a 25–30 Hz beta band power increase thought to represent the final retrieval and activation of the integrated semantic memory, with coactivation of the object‐related representations (Ferree et al., 2009; Slotnick et al., 2002). This pre‐SMA‐thalamus‐caudate circuit is involved during complex, controlled semantic search and retrieval; however, whether this circuit and the neurophysiological metrics (alpha, theta, and beta oscillations) are specific to semantic processing remained an open question at the time.

**(Box 1) cognitive processes during verbal retrieval**

Verbal retrieval is defined here as a general process that indicates getting access to the representation of semantic memory (i.e., concepts and knowledge), words (e.g., lemma, phoneme, and lexicon), or sentence structure (e.g., syntax and prosody). These levels account for different aspects of producing verbal output that is important for successful and efficient verbal communication (for more detailed psycolinguistic and neurolinguistic discussions, see Indefrey & Levelt, 2004; Price, 2012; Vigliocco & Hartsuiker, 2002). Aside from these more classic views of language and speech structure, it has also been proposed that multiple cognitive processes may be involved in this dynamic process to facilitate retrieval (Crosson et al., 2007; Crosson, 2013, 2021; Ries et al., 2016). For example, at the semantic memory level, in order to have a conversation about animals that live in a desert, one ought to initiate a search for such animals based on one's world and conceptual knowledge. Semantic information from multiple related options may be activated simultaneously (e.g., camel, snake, scorpions, lizards, etc.). This would necessitate selection and inhibition processes to eventually narrow down on the correct target (such as a desert animal on which people can ride). This search will continue, and co‐activation of related semantic information will be maintained and reiterated until either selected or inhibited and a single target concept emerges leading to termination of the search and final activation of the integrated targeted concept. This process may be made more challenging depending on the degree of similarity between related concepts, such as discriminating between an alpaca versus a llama or an allegator versus a crocodile (Mahon et al., 2007; Navarette et al., 2014; Oppenheim et al., 2010; Rose et al., 2019; Vigliocco et al., 2002). In other words, increased processing time for search and selection/inhibition may ensue. This can be applied to retrieval at the word or sentence level, and disruption of any of these levels of representation or cognitive processes may lead to deficits in verbal retrieval (Ries et al., 2016; Rohrer et al., 2008).

**(Box 2) experimental tasks**

The Semantic Object Retrieval Test (SORT) is designed to elicit retrieval of semantic memory by presenting cues of features associated with particular objects. In the SORT, two features (as written words) are presented either simultaneously or sequentially for one to decide whether these two features may combine to remind one of a particular object. From example, humps and desert would bring to mind “camel” (retrieval trials), whereas humps and coin would not (non‐retrieval trials) (Assaf, Calhoun et al., 2006; Kraut, Kremen, Segal et al., 2002). A more recent modification, called the multimodal SORT, utilizes features presented in different modalities, where a feature is presented first as a written word, a spoken word, or a picture, followed by a second feature as a written word (Chiang et al., 2016, 2020), whereas one is to make the same type of decision—whether these two features would bring to mind any particular object. The semantic inhibition (SI) tasks are designed to examine selection and inhibition processes during categorization that require semantic processing at multiple levels. In the SI tasks, one responds with a button push to certain stimuli (Go trials) while withholding response to others (NoGo trials). All stimuli are presented as line‐drawing pictures (Brier et al., 2010; Maguire et al., 2009). The semantic complexity of the task is manipulated from one task having only cars for Go and dogs for NoGo to another task having multiple objects including inanimate objects for Go and animals for NoGo. Therefore, these multiple SI tasks require different levels of object categorization depending on the perceptual and semantic complexity of the objects.

**(Box 3) noninvasive electrophysiology and electrical stimulation studies to examine neurocognitive processes**

Scalp EEG, with millisecond resolution, is a noninvasive technique that primarily records the summation of postsynaptic excitatory and inhibitory potentials predominantly from the cortical structures immediately subjacent to the recording electrode. EEG data can be processed to extract event‐related potential (ERP) by averaging multiple trials of EEG data and to extract event‐related spectral perturbation (ERSP) and inter‐trial coherence (ITC) data by performing time‐frequency analysis (Cohen, 2014; Delorme & Makeig, 2004). ERP captures consistent changes in phase‐locked neural activity as reflected in the timing and shape of ERP waveforms (Luck, 2014). ERSP and ITC examine the spectral decomposition of EEG data, which can dissociate differential effects across multiple frequency bands (delta: 1–4 Hz, theta: 4–7 Hz, alpha: 8–12 Hz, beta: 13–30 Hz, gamma: >30 Hz). ERSP indicates magnitude of neuronal activity, whereas ITC indicates the consistency of relative activity (by phase or amplitude, or both) within a neuronal oscillator across trials (Fries, 2015). These different ERP, ERSP, and ITC signatures may be associated with a particular set of neurocognitive processes involved in speech and language functions (Bastiaansen et al., 2005; Etard & Reichenbach, 2019; Indefrey, 2011; Weiss & Mueller, 2012). Noninvasive transcranial electric stimulation (tES) methods include several different techniques that are beyond the scope of our current review (Polania et al., 2018). Generally, these methods deliver electric current via scalp contacts that are thought to reach superficial cortical regions (Huang et al., 2017). In particular, transcranial direct current stimulation (tDCS) is generally thought to modulate cortical neuronal membrane potentials and bias them toward hyperpolarization (cathodal) or hypopolarization (typically anodal), hence modulating cortical excitability acutely while potentially leading to long‐term potentiation and longer‐term effects after repeated stimulation sessions (Filmer et al., 2014; Knotkova et al., 2019). High definition‐tDCS (HD‐tDCS) represents a technical refinement affording improved ability to deliver electrical current to specific brain regions as compared to conventional tDCS (Datta et al., 2009). Previous tDCS studies to enhance verbal retrieval have used different stimulating electrode montages overlying several brain regions to modulate speech and language functions (Monti et al., 2013; Price et al., 2015). Given that EEG is a direct measure of electrical activity from the brain, EEG can be an effective tool to examine changes in neuronal activity secondary to tES modulation (Chiang et al., 2022; To et al., 2018).

## 2 NEURAL DYNAMICS DURING SEMANTIC MEMORY RETRIEVAL: NEWER EVIDENCE FROM SORT AND SI EEG STUDIES AND NONINVASIVE BRAIN STIMULATION STUDIES

### 2.1 New evidence on SORT studies

**FIGURE 2:**
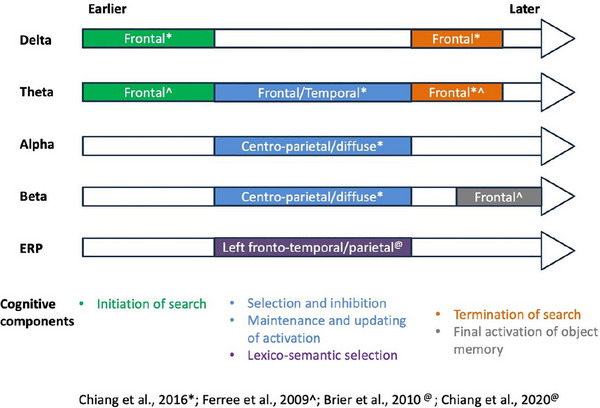
Summary of the TF and event‐related potential (ERP) signatures from the Semantic Object Retrieval Test (SORT) studies. The temporal unfolding of the word retrieval processes is depicted corresponding to each neural oscillatory and evoked activity. (See Text Box 1 for cognitive components of verbal retrieval).

The specificity of the previously proposed semantic memory retrieval neural circuit and its specificity to verbal versus more generally nonverbal semantic retrieval were studied by Chiang et al. (2016, 2020). In this study, object memory using the SORT task was probed by a feature presented as a written word, a spoken word, or a picture, followed by a second feature always presented as a visual word. This design allowed for examination of potential dissociable neural subcomponents (verbal versus nonverbal, visual versus auditory; see Text Box 2) involved in binding object features to retrieve a semantic memory. Across all of the modalities, early event‐related potential (ERP) differences were observed in the left temporal region (350–450 and 500–700 ms) (Chiang et al., 2020). This was proposed to represent early domain‐general and modality‐independent integration or activation of semantic information where feature pairs start to bind or the processes associated with earlier stages of word search/word retrieval processes as part of the overarching lexico‐semantic retrieval. Modality‐specific activity was also identified, including a left frontotemporal ERP (750–1100 ms poststimulus onset) in versions using verbal (visual and auditory words) stimuli but not in nonverbal stimuli (picture). These results suggest that lexico‐semantic selection indexed by the left frontotemporal ERP leads to activation of an integrated object memory in retrieval trials primarily facilitated by verbal but not nonverbal information (see Figure 2 ). There was also right frontotemporal ERP activity found only in the auditory word version (850 and 1000 ms poststimulus onset), designating engagement of the auditory aspects of the verbal semantic subsystem.

**FIGURE 3:**
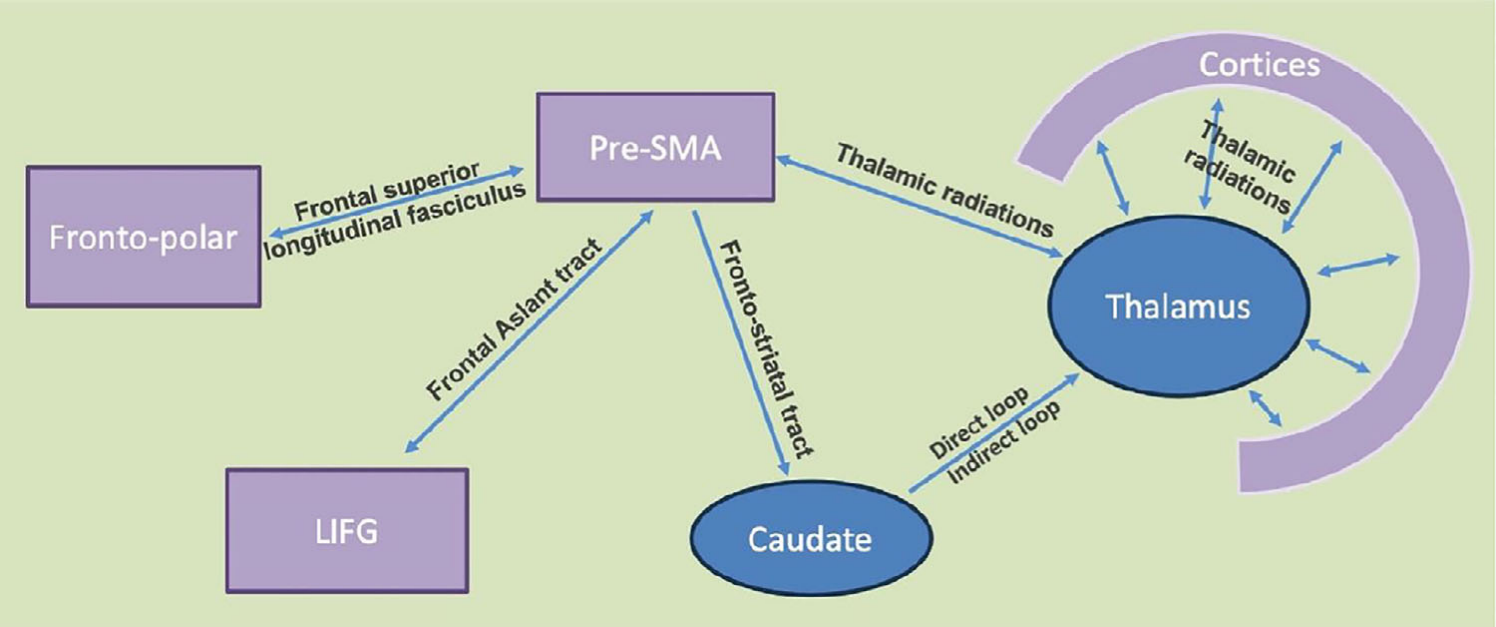
Structural connectivity between regions within the circuit. Purple shade represents cortical regions; blue shade represents subcortical regions. LIFG, left inferior frontal gyrus; Pre‐SMA, pre‐supplementary motor area.

Time‐frequency analysis using the same multimodal SORT (Chiang et al., 2016; Text Box 2) further elucidated the retrieval mechanism. Across all modalities in retrieval compared to non‐retrieval trials, there was greater delta synchronization (in the frontal regions), theta desynchronization (in the fronto‐central and temporal regions), and alpha/beta desynchronization (in centro‐parietal, more diffuse) time‐locked to stimulus onset. Other than delta effects that peaked around 500 ms poststimulus onset, effects in theta/alpha/beta persisted across a protracted time course between 600 and 1200 ms poststimulus onset. Given the temporal unfolding of these oscillatory activities, delta synchronization at early stages is thought to reflect initiating search during retrieval, similar in spatiotemporal distribution to what was previously reported as early increased theta synchronization (200–300 ms poststimulus onset; in Ferree et al., 2009). In contrast, the increased theta desynchronization later, along with alpha and beta desynchronization, may indicate gaining access to cortical representations of object memory and integration of semantic information leading to object memory retrieval, supported by their prolonged time course lasting from 600 to 700 ms until 1200 ms poststimulus (about the average response time) (Figure 2). Alpha desynchronization has been associated with suppression of global processing that facilitates task‐based regional activation (Li & Yang, 2013; Pfurtscheller & Lopes Da Silva, 1999; Shahin et al., 2009) and successful object activation in memory (Bartsch et al., 2015; Michel et al., 1994). Beta band EEG power change has been linked to lexical or semantic retrieval (Bakker et al., 2015; Bastiaansen et al., 2008; Lewis et al., 2015; Slotnick et al., 2002). Furthermore, prior to subjects making a response (peaking around 200–400 ms prior to response), there was increased delta and theta synchronization in the frontal regions during retrieval trials compared to non‐retrieval trials. This was proposed to be associated with controlled processes during retrieval and termination of search before making a decision (Figure 2). Delta band activity has been associated with cortical inhibition in the medial frontal cortical regions proposed to attenuate irrelevant activity for the current task (Harmony, 2013), whereas theta band activity has been linked to cognitive control functions during memory retrieval (Bakker et al., 2015; Mellem et al., 2013; Shahin et al., 2009). We previously posited that theta power changes related to cognitive control could reflect information accumulation and integration occurring prior to or around making a decision needed for memory retrieval (Werkle‐Bergner et al., 2014). Though not evident in the multimodal SORT studies, prior studies using SORT (Ferree et al., 2009; Slotnick et al., 2002), have found a late beta activity (around 25–30 Hz) around 1100 ms poststimulus onset indicating corticothalamic activity linked to final activation of object memory retrieval (Figure 2). It is plausible that the difference in beta was due to the difference in simultaneous stimulus presentation (in Ferree et al., 2009; Slotnick et al., 2002) versus sequential stimulus presentation (Chiang et al., 2016).

These electrophysiological measures provide excellent temporal resolution down to milliseconds of temporal unfolding to indicate the sequences of neurocognitive processes during semantic memory retrieval, but only approximation in localization (e.g., frontal, temporal, centro‐parietal regions, etc.). Still, there is supporting evidence from neuroimaging studies to indicate the potential sources of these separate neurocognitive activities, including frontal activity for initiation and termination of search (Becker et al., 2020; Li et al., 2017; Olivers et al., 2005; Pollmann et al., 2000), frontal activity for selection and inhibition (Goghari & MacDonald, 2009; Maril et al., 2001; Mostofsky & Simmonds, 2008; Rae et al., 2014), frontal and more diffuse centro‐parietal activity for maintenance and updating of activation (Pollmann et al., 2000; Shahdloo et al., 2022; Emch et al., 2019), left frontotemporal activity for lexico‐semantic selection (Thompson‐Schill et al., 1997, 1999; Wagner et al., 2014), as well as frontal activity in final activation and retrieval of memory (Assaf et al., 2009, 2006; Hayes et al., 2004). Notably, although many of these processes are central to semantic processing, they may not be specific to semantic memory function.

### 2.2 New evidence from SI studies

The data from the SI studies show that theta power increases in two distinct regions—one at the midline frontal pole region and the other overlaying the pre‐SMA area, for the NoGo stimuli compared to the Go stimuli (Brier et al., 2010). This neural activity in both the pre‐SMA and the fronto‐polar regions is modulated by semantic information, with reduction in theta power corresponding to increased semantic complexity during object categorization (from distinguishing dogs versus cars to animals versus nonanimal objects). There is also increased theta connectivity (i.e., coherence) between these two regions in NoGo compared to Go. This pre‐SMA‐frontal polar theta power activity was previously proposed by Hart et al. (2013) to indicate an inhibitory mechanism during semantic memory retrieval. More recently, DeLaRosa et al. (2020b) found that dominant generators of theta‐band EEG activity were localized near the left pre‐SMA and right thalamus, as well as the midline fronto‐polar region during a Go/NoGo task. These theta band power changes best distinguished NoGo trials (inhibiting a distractor item) from Go trials (selecting a correct target), suggesting important roles of neural processes represented by theta brain rhythms in selecting and inhibiting potentially competing information when identifying a target object in semantic memory.

To localize regional brain activity during the SI task, Chiang, Motes et al. (2013) used functional MRI. Multiple brain regions were found to be involved in selection and inhibition during object identification and categorization, particularly near the pre‐SMA region (BA6 and BA32) corroborating findings of our EEG studies, and suggesting that the pre‐SMA might be the source of these electrical signals (i.e., N2/P3, frontal theta).

### 2.3 HD‐tDCS studies targeting the pre‐SMA

Noninvasive brain stimulation may serve as an effective tool to test if modulation of the pre‐SMA leads to changes in brain activity and behavioral performance during retrieval of semantic memory (see Text Box 3). Using anodal high‐definition tDCS (HD‐tDCS) targeting the pre‐SMA, one study (To et al., 2018) found behavioral improvement in a classic Stroop task (faster in inhibiting incongruent trials), along with corresponding increased beta activity within the pre‐SMA and fronto‐polar regions, indicating improved inhibition mediated by the pre‐SMA and fronto‐polar activity. DeLaRosa et al. (2020a)used HD‐tDCS targeting the pre‐SMA and found that a larger frontal N2 amplitude (related to selection and inhibition functions) was induced for NoGo after stimulation compared to sham stimulation. In addition, response times were faster for Go trials during the SI task, indicating an important role of the pre‐SMA in both selection and inhibition during retrieval processes. There is further evidence supporting the role of pre‐SMA in inhibition and selection in retrieval from studies conducted using HD‐tDCS involving chronic TBI patients with verbal retrieval deficits. A study by Motes et al. (2020) found that 10 sessions of 20‐min anodal HD‐tDCS targeting the pre‐SMA in TBI patients resulted in sustained improved category fluency. In examining changes in neural activity subsequent to pre‐SMA HD‐tDCS immediately and 6 weeks after 10 sessions of active treatment, Chiang, Shakal et al. (2021) found reduced latency and increased amplitude of the frontal N2 during the SI task, as well as improved reaction time and inhibitory response compared to baseline in a patient with chronic cognitive sequelae of severe TBI. Later, a study of a group of military combat veterans with chronic TBI found increased frontal theta/delta power and phase coherence (within the frontal regions) during the SI task immediately after 10 sessions of pre‐SMA stimulation (Chiang, Motes et al., 2022). Pre‐SMA stimulation using either conventional tDCS or HD‐tDCS has also been used by other research groups and showed potential modulatory effects on improving selecting and inhibitory functions in healthy and disease states (Guo et al., 2022; Hsu et al., 2011; Khan et al., 2022; Pathak et al., 2022). These neuromodulation/tDCS studies further suggest an important role of the pre‐SMA in the retrieval circuit for selection and inhibition during search.

## 3 A MODIFIED NEURAL CIRCUIT FRAMEWORK OF SEMANTIC MEMORY RETRIEVAL

We propose that the retrieval circuit maintains its coordinated activity amongst its components as well as with other regions outside the main circuit via oscillatory electrical activity. Activity in different frequency bands and at different times index different functions within the circuit related to initiation and termination of search, selection/inhibition of related and similar or competing information, integration and generation of coherent information, and eventually retrieval of integrated, multi‐semantic subsystem object/concept activation and subsequent word retrieval.

Some neural activity is likely domain/modality‐general and involved in retrieval processes independent of stimulus types. These include early frontal theta and delta band synchronized activity, which appears to be generated in the pre‐SMA and is involved in initiation of search and selection/inhibition during retrieval processes (Figures 1 and 2). This early theta activity is coordinated through increased coherence between the pre‐SMA and fronto‐polar regions, corresponding to frontal N2/P3 components, to facilitate inhibition (Figure 1). Frontal pre‐SMA theta/delta activity can be modulated, as indicated by increased frontal theta/delta power and phase coherence by targeting the pre‐SMA using HD‐tDCS. In contrast, later frontal theta desynchronized activity lasting over a few 100 ms (600–1100 ms post stimulus onset) indicates continuous selection and inhibition of competing information (Figure 2), which is also coordinated within (caudate and thalamus) and external to the circuit (other cortical regions) through alpha and beta desynchronized activity across overlapping epochs (Figure 1). This theta desynchronized activity, overlapping with the time course of alpha and beta desynchronization, may indicate continuous selection and inhibition during search and may be distinguished from other theta activities at different time frames, as there is an early theta more dominant in initiation and a later theta (close to response/decision) linked to termination of search (Figure 2). We also propose that, in comparison to frontal theta‐band EEG activity, more diffusely distributed (over the centro‐parietal regions) beta‐band EEG and alpha‐band EEG activities starting around 750 ms involve a thalamocortical circuit and cortical suppression, respectively, while together are indicative of integration, co‐activation of multiple systems, and maintenance of lexico‐semantic representations being retrieved (Figures 1 and 2). This early beta is also distinguished from later beta activity peaking about 1100 ms at 25–30 Hz that signifies final activation of object memory (Ferree et al., 2009; Slotnick et al., 2002). These differences in oscillatory activity across multiple frequency bands corresponding to the earlier fronto‐parietal evoked potentials indicate potentially domain‐general and modality‐independent processes involving the retrieval circuit (Figures 1 and 2).

The interaction between the pre‐SMA and the fronto‐polar region is thought to indicate domain‐general inhibitory function (Figure 1). The frontal polar regions monitor and select the action of inhibition, whereas the pre‐SMA is more directly engaged in inhibiting the motor response. EEG coherence connectivity analysis indicated increased theta interactions between the frontal polar and the pre‐SMA regions in the NoGo versus Go conditions, supporting the notion that the frontal pole interacts with the pre‐SMA while engaged in inhibition (Rubia et al., 2001). Lesions of the frontal pole have been shown to increase reaction times substantially in Go/NoGo tasks, and lesions of pre‐SMA areas have been shown to increase the number of commission errors in the NoGo condition (Picton et al., 2007). Although lesion studies do not directly specify the cognitive operations underlying the neural activity in the frontal polar region, functional imaging studies have implicated this region in action selection, response inhibition, and performance monitoring (Ridderinkhof et al., 2004), all of which are plausible in this setting.

Compared to the abovementioned neural processes that appear to be more domain‐general and modality‐independent, certain neurodynamic changes during retrieval show more selectivity for certain domains and modalities. For example, the later left frontotemporal evoked potential during semantic memory retrieval is stronger when retrieval is based primarily on verbal information compared to addition of nonverbal information. This left frontotemporal activity has been shown to correlate with verbal fluency measures (Chiang et al., 2015). We propose this may reflect activity in the left inferior frontal gyrus (LIFG) because of its spatial distribution. Based on EEG activity, we contend that verbal semantic information may require additional recruitment from the LIFG, which is more domain specific to lexico‐semantic processing (Price, 2012; Tremblay & Gracco, 2006; Wagner et al., 2014), although both verbal and nonverbal information require the pre‐SMA‐caudate‐thalamus circuit, which appears to be more domain general and modality independent, during retrieval of semantic memory. We will discuss in greater detail on the role of LIFG in retrieval, compared to the pre‐SMA, in a later section.

## 4 THE PRE‐SMA, STRIATAL, THALAMIC CIRCUIT IS ALSO CENTRAL TO WORD RETRIEVAL AND SPEECH PRODUCTION

Thus far we have focused on the neural circuit consisting of pre‐SMA, basal ganglia (e.g., caudate), and thalamus in mediating the semantic memory retrieval functions. This retrieval process in semantic memory shares overlapping characters with verbal production (see Text Box 1). Neural mechanisms of selecting the correct and relevant representations while inhibiting the incorrect and irrelevant representations during word retrieval and speech production bear important implications to understanding retrieval deficits in patient populations and to devising novel treatment strategies. In this next section, we will attempt to focus on relevant evidence strongly supporting the role of the pre‐SMA‐striatal‐thalamic circuit in word and speech (verbal) production.

### 4.1 Role of pre‐SMA, basal ganglia, and thalamus in verbal production

The pre‐SMA is central to word retrieval and speech production, based on both normal and patient studies (Hertrich et al., 2016; Lu et al., 2021; Wagner et al., 2014). The pre‐SMA is distinguished from the SMA not only anatomically but also functionally. The pre‐SMA is found to coordinate, initiate, and inhibit elements of speech execution such as syntax and prosody, as well as to transfer semantic information into speech programing (Anders et al., 2019; Hertrich et al., 2016; Nachev et al., 2008), serving as an interface between other cortical and subcortical (e.g., anterior thalamus, caudate, dentate nucleus of the cerebellum) regions. The role of the pre‐SMA has been examined using the “artificial,” temporary, lesion‐inducing technique of transcranial magnetic stimulation and is found to be more specifically involved in voluntary word and speech production when volitional word production is compared to forced word production (Tremblay & Gracco, 2009). Correlational studies based on fMRI BOLD signal also demonstrate more activation in the pre‐SMA in voluntary speech (generating a word compared to reading a word in Tremblay & Gracco, 2006; volitional word production compared to forced word production in Tremblay & Gracco, 2010) and speech‐specific production (word production requiring selection compared to word production without selection in Alario et al., 2006). Impaired verbal fluency has been linked to lesions or dysfunction of the pre‐SMA in patients with stuttering, stroke, or neurodegenerative diseases such as progressive supranuclear palsy (Busan, 2020; Isella et al., 2022; Obayashi, 2020). Surgical removal or ischemic damage of the SMA/pre‐SMA with neighboring tissues may lead to SMA syndrome, characterized by reduced fluency to the point of speech arrest (Pinson et al., 2022; Potgieser et al., 2014).

Nuclei in the basal ganglia and thalamus (typically on the left) that facilitate speech production have stronger functional and structural connections to the pre‐SMA (Lou et al., 2017; Ruan et al., 2018). In particular, lesions in the pulvinar and anterior/ventrolateral thalamic nuclei have been associated with deficits in lexical‐semantic retrieval (as reported in thalamic aphasia; Fritsch et al., 2022; Radanovic & Almeida, 2021; Segal et al., 2003). Pathophysiologically, the deficits may be the result of more direct interference with thalamic function (Crosson, 2013, 2021; Wang et al., 2021) or indirect impact via diaschisis mediated by cortical hypoperfusion/hypometabolism (e.g., pre‐SMA hypoperfusion in Fritsch et al., 2022; Obayashi, 2020). These thalamic nuclei are central for relaying and integrating information streams between multiple cortical regions, as well as selecting and engaging relevant on‐line lexico‐semantic information for word production (see Crosson, 2013, 2021). In comparison to the thalamus, the basal ganglia (especially striatum, i.e., caudate and putamen) may have a role in speech and language production involving sequencing and procedural learning that is not specific to speech and language (Copland et al., 2021; Radanovic & Almeida, 2021; Shi & Zhang, 2020). Multiple brain rhythms recording from the basal ganglia (e.g., subthalamic nucleus) have been implicated as the electrophysiological basis for verbal production (alpha–theta rhythms in Wojtecki et al., 2017; gamma activity in Anzak et al., 2011). Lesions in basal ganglia can be associated with different patterns of speech and language symptoms (Jacquemot & Bachoud‐Lévi, 2021; Radanovic & Almeida, 2021), with the caudate nucleus in particular found to be involved when there is increased cognitive demand in control and selection for speech production and language processing (Copland et al., 2021; Jacquemot & Bachoud‐Lévi, 2021; Robles et al., 2005; Thames et al., 2012). Thalamic dysfunction is more characterized by phenotypical aphasia syndromes, while basal ganglia dysfunction typifies more subtle and executive dysfunction in word retrieval and speech production (Crosson et al., 2007; Crosson, 2021; Ehlen et al., 2014; Hebb & Ojemann, 2013; Kim et al., 2021; Radanovic & Almeida, 2021; Radanovic & Scaff, 2003; Tiedt et al., 2017, 2021).

### 4.2 Interrupted connections within the neural circuit can disrupt verbal production

The left frontal aslant tract (FAT) connects the SMA complex to LIFG (including pars triangularis and pars opercularis) and has been shown to play an important role in speech production and verbal fluency (Catani et al., 2013; Ford et al., 2010). Studies using interoperative direct electrical stimulation and/or postoperative tractography based on DTI demonstrated interruption of the left FAT has been associated with reduced verbal fluency (Chernoff et al., 2019, 2018; Fujii et al., 2015; Kinoshita et al., 2015; Sierpowska et al., 2015; Vassal et al., 2014), motivating pre‐operative planning to avoid lesions in FAT to minimize such deficits (Briggs et al., 2021). Interruption of the left FAT using direct electric stimulation was shown to reduce voluntary speech fluency or sentence completion but did not affect sentence repetition or picture naming (Chernoff et al., 2018; Dragoy et al., 2020). Damage to or abnormal development of the FAT (most often left lateralized) is associated with verbal dysfluency, reduced speech initiation and stuttering, non‐fluent/agrammatic variant primary progressive aphasia (nfvPPA), and developmental stuttering (Catani et al., 2013; Cipolotti et al., 2020; Costentin et al., 2019; Kemerdere et al., 2016; Keser et al., 2020; Kronfeld‐Duenias et al., 2016; Mandelli et al., 2014, 2016). Disruption of this tract may also lead to SMA syndrome characterized by reduced or arrested speech production (Agyemang et al., 2023; Pinson et al., 2022). Thus, the FAT has been shown to be involved in verbal fluency, which encompasses initiation and volition of speech production, while simple naming and reading may not be affected by disruption of the FAT.

The pre‐SMA is also structurally connected to the striatum in the basal ganglia (i.e., caudate and putamen) via the fronto‐striatal tract (FST, part of the subcallosal fasciculus) (Bozkurt et al., 2016; Lehéricy et al., 2004) and to the thalamus via the fronto‐thalamic radiations (Inase, 1996). FST involves cortico‐basal ganglia pathways that facilitate cognitive control in selecting and inhibiting among multiple motor/speech programs during speech production important for fluency (Dick et al., 2019; Kinoshita et al., 2015). Disruption or impaired integrity of the left FST is associated with reduced verbal fluency and spontaneous speech, whereas simple motor reading is not necessarily affected (Duffau et al., 2002; Catani et al., 2013; Costentin et al., 2019; Dresang et al., 2021; Kinoshita et al., 2015; Mandelli et al., 2014; Rodriguez‐Porcel et al., 2021). Disruption in the left FST has also been associated with nonfluent aphasia due to stroke and nfvPPA (Dick et al., 2019; Mandelli et al., 2014). Though evidence is preliminary and somewhat limited, disruption in the corticothalamic and thalamocortical tracts (e.g., anterior thalamic radiations) may also be associated with impaired verbal production and naming possibly due to abnormal lexico‐semantic processing secondary to disconnection between thalamus and frontal cortical regions (Cipolotti et al., 2020; Dresang et al., 2021; Kim et al., 2021). Studies have shown altered functional connectivity between the pre‐SMA and thalamus associated with verbal retrieval deficits (Qiao et al., 2017), which may be due to altered direct versus indirect connectivity. Thus, both the FST and tracts connecting the thalamus to the cortical regions are suggested to be central to verbal fluency and voluntary verbal production, likely by connecting the pre‐SMA to the striatum and thalamus.

### 4.3 A modified neural circuit framework of semantic memory retrieval—connecting the dots: structural connectivity in the retrieval circuit (Figure 3)

As was previously hypothesized in Hart et al. (2013), disconnection in the retrieval circuit, especially between the pre‐SMA and other regions central for verbal retrieval, would be predicted to disrupt retrieval of information. Although these have not been directly addressed based on the SORT and SI tasks, the reviewed literature we have presented supports the importance of the pre‐SMA, striatum (caudate), and thalamus in verbal retrieval and speech production, which constitutes a shared retrieval circuit with semantic memory retrieval.

Retrieval of words and semantic information is fundamental to verbal production and requires similar processes that include search, selection, and inhibition; maintenance and integration of the activated semantic information distributed across multiple semantic subsystems; and finally, coherent activation of the aimed integrated semantic object memory representation and subsequently the name associated with that object. Structural connectivity between the pre‐SMA and other regions (e.g., FAT to LIFG, FST to the striatum, thalamic radiations from and to the thalamus), as parts of the retrieval circuit, is vitally important to coordination of activity across brain regions. Disruption of these connecting white matter tracts results in impaired verbal retrieval, potentially due to reduction in coordinated and synchronized neural activity across these regions. Of note, connection from the pre‐SMA to the fronto‐polar regions is likely through the frontal subcortical short association “U” fibers and the frontal superior longitudinal fasciculus system (Catani et al., 2013), that is, likely not specific to verbal retrieval function but is linked to domain general inhibitory and monitoring functions as suggested by studies examining the fronto‐polar regions (Picton et al., 2007; Ridderinkhof et al., 2004; Rubia et al., 2001).

### 4.4 A modified neural circuit framework of semantic memory retrieval—evidence supporting a role of LIFG in the retrieval circuit (Figures 1 and 2)

It has been long known that the LIFG plays a central role in lexical semantic processing (Price, 2012; Tremblay & Gracco, 2006; Wagner et al., 2014). In general, the more anterior aspect of the LIFG (pars triangularis) is more selectively involved during selection operations of semantic processing, whereas the more posterior part (pars opercularis) is more involved during selection in lexical/phonological and syntactical processing based on a multitude of correlational, direct ES, and lesion‐deficit studies (Chang et al., 2018; Leonard et al., 2019; Lu et al., 2021; Price, 2012; Thompson‐Schill et al., 1997, 1999).

FMRI signal change in the LIFG in subjects performing the SORT was also found in the retrieval condition during object memory activation. Kraut, Kremen, Moo et al. (2002) showed increased BOLD signal in both the left pre‐SMA and the LIFG during a multimodal SORT and Assaf, Calhoun et al. (2006) showed more activation in the LIFG for SORT but not in a semantic association control task. Additionally, Assaf, Rivkin et al. (2006) showed activation of the LIFG for retrievals in both normal controls and schizophrenic patients. Assaf et al. (2009), assessing functional connectivity of task‐related BOLD in healthy subjects, delineated a left hemisphere network encompassing both the LIFG and pre‐SMA, independent from the pre‐SMA‐thalamus network. Calley et al. (2010), studying Gulf War Illness patients, found that the LIFG was also activated during semantic memory retrieval. Whether activation of the LIFG in semantic memory retrieval in these studies reflects covert naming, hence reflecting lexical and semantic selection (Price, 2012; Thompson‐Schill et al., 1997; Hirshorn & Thompson‐Schill, 2006), or is central to integrating semantic information is unclear.

Anatomically, overlapping neural circuits, including the pre‐SMA‐caudate‐thalamus, as well as the LIFG, are identified in both semantic memory and word retrieval. Theoretically, selection and inhibition of competing information are central to both word and semantic memory retrieval. Retrieval processes may be closely and quickly coordinated between the pre‐SMA (caudate‐thalamus circuit) and the LIFG, as indicated by the established white matter tract, FAT, with monosynaptic signal transmission connecting these two regions (Ookawa et al., 2017). We propose that the LIFG communicates via FAT with the pre‐SMA‐caudate and thalamus circuit predominantly when verbal processing is necessitated, even during processing of the cadence and emotional features of speech such as in the case of prosody generation and perception (Pichon & Kell, 2013; Rovetti et al., 2022; Sammler et al., 2015). This sheds light on potential functional differentiation between the LIFG and pre‐SMA during verbal retrieval. We posit that the LIFG has a central and strategic role in more detailed processing of lexico‐semantic information, given its connection to the temporal systems via the arcuate fasciculus and the inferior fronto‐occipital fasciculus (Dick & Tremblay, 2012; Turken & Dronkers, 2011). On the other hand, the pre‐SMA may be the central mediator to initiate and terminate processing of voluntary retrieval facilitated by striatum (e.g., caudate) and thalamus in the context of increased cognitive demand. It is also plausible that the pre‐SMA‐striatal‐thalamic circuit coordinates both declarative and procedural aspects of verbal retrieval (Copland et al., 2021; Crosson, 2021; Jacquemot & Bachoud‐Lévi, 2021), intentional control (Zapparoli et al., 2018), as well as conflict resolution (Goghari & MacDonald, 2009; Mayer et al., 2012), which appears to lie beyond the role of the LIFG during verbal retrieval. There is a right‐lateralized system (with right FAT and right FST) central to motor response inhibition that mirrors the left‐lateralized system central to language/speech production (Dick et al., 2019). This right‐lateralized system includes both the right pre‐SMA and right inferior frontal gyrus, and the functional associations between these two regions have been examined primarily for motor inhibition (Guo et al., 2022; Nachev et al., 2008; Obeso et al., 2013; Xu et al., 2016). If and how this right lateralized system, central to motor/response inhibition, interacts with the left lateralized system remains to be studied.

### 4.5 A modified neural circuit framework for semantic memory retrieval—findings from the disease states (see summary in Table 1)

Both lesions to a region (pre‐SMA, thalamus, striatum, LIFG) and lesions to a connection (FAT, FST, thalamic radiation, frontal short U fibers) can affect verbal retrieval functions, as we have reviewed. Our currently updated neurocognitive circuit framework would further predict that (1) lesion to or dysfunction of a particular region in the retrieval circuit can impact oscillatory/evoked brain activity and (2) such change in the oscillatory/evoked brain activity can impact verbal retrieval functions via different cognitive subcomponents that could be domain‐general versus domain‐specific.

More specifically, we predicted that lesion to or dysfunction of the pre‐SMA can affect frontal evoked and oscillatory activity and results in verbal retrieval deficits. These deficits would be more domain‐general with impairment in search, impaired selection and inhibition of competing information as the core dysfunction during verbal retrieval, and impaired ending of search and synchronizing representations to form the integrated object memory. There are some preliminary findings that have supported this prediction. In a pilot study using fMRI during the SORT, it was found that amnestic MCI patients had reduced regional activation during retrieval trials in the medial aspects of both frontal lobes (part of the pre‐SMA near BA6 and BA32), suggesting this retrieval circuit was affected by the disease state, resulting in decreased retrieval accuracy (Chiang, Mudar et al., 2013). It was also found that frontal theta power during NoGo and parietal alpha power during Go were reduced in MCI patients, suggesting reduced neural activity in both selection and inhibition processes during retrieval, respectively (Nguyen et al., 2017). In schizophrenia patients who tended to produce more false positive answers to non‐retrieval trials (“overrecalled trials”) during the SORT, hyperactivation was found within the pre‐SMA, thalamus, and caudate as well as over‐recruitment of the right lateralized semantic systems, compared to controls (Assaf et al., 2006).

We also predicted that lesion to or dysfunction of the LIFG can affect retrieval of word and verbal information and the left frontotemporal ERP component, whereas retrieval of nonverbal semantic information may still remain more preserved. This indicates more domain‐specific, rather than domain‐general, deficits. Based on our preliminary findings, patients with MCI compared to normal controls showed increased left frontotemporal/parietal ERP activity in the 950–1050 ms poststimulus onset (Chiang et al., 2015). This left frontotemporal/parietal ERP during non‐retrieval trials was negatively correlated with letter fluency performance (i.e., total number of words generated in a minute that start with F, A, and S) across both normal controls and MCI. Accuracy was significantly reduced across both retrieval and non‐retrieval trials in MCI compared to normal controls, indicating impaired retrieval performance. In veterans with Gulf War Illness who also demonstrated reduced retrieval accuracy during the SORT, there was an accentuated late fronto‐parietal ERP component around 750 ms, similar to MCI patients, when compared to normal controls (Tillman et al., 2017). In contrast, Fratantoni et al. (2017) reported reduction in the left frontotemporal ERP component in retired NFL athletes with prior repetitive concussions who experienced more verbal retrieval difficulties in older age, compared to age‐matched normal controls.

We further predicted that lesions in or dysfunction of the caudate and thalamic nuclei can affect theta, alpha, and beta rhythms, leading to more difficulty in maintaining and updating of co‐activation of multiple cortical regions and subsystems causing slowed or impaired retrieval functions. In addition, disruption of late beta may lead to disruption of final activation of the targeted concept or word causing failure in retrieval. Although we do not have direct evidence, data on patient populations have shown preliminary support. SORT fMRI experiments showed changes in patterns of activation within caudate and thalamus in Gulf War veterans (Calley et al., 2010) and reduced regional activation in the left caudate during retrieval trials in MCI (Chiang, Mudar et al., 2013). Both patient populations demonstrated more verbal retrieval difficulty compared to controls.

Importantly, our neurocognitive framework predicted that modulating regions that mediate such oscillatory/evoked brain activity could potentially improve verbal retrieval functions. For example, modulating theta and delta activity within the pre‐SMA would be predicted to improve verbal retrieval function. In fact, our recent study (Chiang et al., 2022) has shown that pre‐SMA modulation using HD‐tDCS led to increased power and intertrial phase coherence of the frontal theta and delta‐band EEG activity, and baseline frontal theta power and coherence in particularly predicted improvement in response time and accuracy during response selection in the SI task.

## 5 A NOVEL PATHWAY FOR INTERVENTION OF VERBAL RETRIEVAL DEFICITS

With the advancement of noninvasive stimulation techniques, more precise modulation of the retrieval circuit, such as the pre‐SMA and/or LIFG, may improve our understanding of their differential roles in verbal retrieval, providing different strategies to improve verbal retrieval deficits. Reduced or impaired verbal retrieval performance in neurologic populations may provide windows of opportunity to examine these questions and potential applications. We have focused on modulating the pre‐SMA as the target to potentially modulate the neurodynamics of the circuit to improve memory and word retrieval function in normal and patient populations. It will be critical not only to show that this intervention can be effective in treating verbal retrieval deficits, as has been reported recently (Chiang et al., 2022, 2021; Chiang, Fratantoni et al., 2022; Motes et al., 2020), but also to predict individual responsiveness to the stimulation protocol, which will help select more appropriate candidates to receive such treatment (Chiang, Motes, et al., 2022, 2021). In our recent study designed to examine baseline predictive factors, we separated individuals with intact versus impaired baseline delayed verbal recall as a proxy for frontal and temporal lobe integrity (Chiang, Motes, et al., 2021). TBI patients with impaired baseline delayed verbal recall had significant improvement in verbal retrieval (category fluency) in response to active pre‐SMA HD‐tDCS compared to sham. This implies that aspects of the HD‐tDCS response are subject to the underlying functional and structural brain integrity. As some studies have shown the value of using baseline structural measures in predicting responses to tDCS (de Aguiar et al., 2020; Zhao et al., 2021), we hypothesize that the integrity of the retrieval circuits, contingent upon the structural connectivity via the major white matter tracts (e.g., FAT, FST, and anterior thalamic radiations), may constrain the extent of interventional effects. In addition to structural connectivity, baseline functional connectivity measured directly by EEG or indirectly by fMRI may also be predictive of potential benefits of the modulation effects of the circuit. Future studies testing these hypotheses can not only further mechanistic understanding of the retrieval circuit but also provide novel strategies to optimize neuromodulation applications in neurologic patients with verbal retrieval deficits.

## 6 CONCLUSION

In this review, we elaborate on a modified and updated neural circuit framework of verbal and semantic memory retrieval based on synthesis and integration of more recent neurophysiological and neuroanatomical evidence. The evidence is based on studies using normal subjects and clinical populations where verbal retrieval deficits can be prominent. We contend that a shared retrieval circuit is central for both semantic memory retrieval in general and word production in specific, and that this circuit involves the pre‐SMA, basal ganglia (particularly caudate), thalamus, as well as the fronto‐polar regions and LIFG as the primary sites that are closely connected to and centered around the pre‐SMA. We emphasize that disconnection between these regions, or desynchronized activity among these regions even without apparent structural disconnection, may lead to verbal retrieval deficits. Future studies will need to focus on examining both functional and structural connectivity between these regions to understand better the normal function of this circuit as well as how it is impacted in disease states that may lead to deficits. Although verbal retrieval deficits remain a challenging symptom to treat in clinical populations, studies have shown promise in modulating the neural circuit to improve synchronized neural activity within the retrieval circuit and remediate verbal retrieval deficits.

## AUTHOR CONTRIBUTIONS

**Hsueh‐Sheng Chiang**: Conceptualization; writing—original draft; writing—review and editing; visualization; supervision; funding acquisition; investigation. **Raksha A. Mudar**: Writing—original draft; writing—review and editing; visualization. **Christine S. Dugas**: Writing—review and editing. **Michael A. Motes**: Writing—review and editing. **Michael A. Kraut**: Writing—review and editing. **John Hart**: Writing—original draft; writing—review and editing; conceptualization; supervision; visualization; investigation.

## CONFLICT OF INTEREST STATEMENT

The authors declare no conflicts of interest.

### PEER REVIEW

The peer review history for this article is available at https://publons.com/publon/10.1002/brb3.3490.

7

**TABLE 1:** Predictions based on the neural circuit framework and early empirical evidence based on different disease states.

Principles	Predictions	Empirical evidence
Lesion to or dysfunction of a particular region in the retrieval circuit can impact oscillatory/evoked brain activity	Pre‐SMA: more domain‐general with impairment in search, impaired selection and inhibition of competing information as the core dysfunction during verbal retrieval, and impaired ending of search and synchronizing representations to form the integrated object memory	Reduced pre‐SMA activation during retrieval trials in amnestic MCI patients during SORT (Chiang, Mudar, et al., 2013)
		Reduced frontal theta power during NoGo and parietal alpha power during Go in MCI patients, suggesting reduced neural activity in both selection and inhibition processes during retrieval (Nguyen et al., 2017)
Change in the oscillatory/evoked brain activity can impact verbal retrieval functions via different cognitive subcomponents		Hyperactivation within the pre‐SMA in schizophrenia patients who tended to produce more false positive answers to non‐retrieval trials during SORT (Assaf et al., 2005)
		Pre‐SMA modulation using HD‐tDCS led to increased power and intertrial phase coherence of the frontal theta and delta‐band EEG activity (Assaf et al., 2005).
	LIFG: more of domain‐specific, rather than domain‐general, deficits during retrieval of word and verbal information	Increased left frontotemporal/parietal ERP activity in amnestic MCI during SORT (Chiang et al., 2015)
		An accentuated late fronto‐parietal ERP component during SORT around 750 ms in veterans with Gulf War Illness (Tillman et al., 2017)
		Reduced left frontotemporal ERP component during SORT in retired America football athletes with who experienced verbal retrieval difficulties (Fratantoni et al., 2017)
	Caudate/Thalamus: lesions in or dysfunction of the caudate and thalamic nuclei can affect theta, alpha, and beta rhythms, leading to more difficulty in maintaining and updating of co‐activation of multiple cortical regions and subsystems causing slowed or impaired retrieval functions	Altered activation within caudate and thalamus in Gulf War veterans with verbal retrieval deficits during SORT (Calley et al., 2010)
		Reduced regional activation in the left caudate during retrieval trials in SORT in MCI (Chiang, Mudar, et al., 2013)
		Hyperactivation within the thalamus and caudate in schizophrenia patients who tended to produce more false positive answers to non‐retrieval trials during SORT (Assaf et al., 2005)

Abbreviations: EEG, electroencephalography; ERP, event‐related potentials; HD‐tDCS, High definition transcranial direct current stimulation; LIFG, left inferior frontal gyrus; MCI, mild cognitive impairment; Pre‐SMA; pre‐supplementary motor area; SORT, semantic object retrieval task.

## Data Availability

Not applicable.
